# Integrated Metabolomics-DNA Methylation Analysis Reveals Significant Long-Term Tissue-Dependent Directional Alterations in Aminoacyl-tRNA Biosynthesis in the Left Ventricle of the Heart and Hippocampus Following Proton Irradiation

**DOI:** 10.3389/fmolb.2019.00077

**Published:** 2019-09-10

**Authors:** Eileen Ruth S. Torres, Reed Hall, Gerd Bobe, Jaewoo Choi, Soren Impey, Carl Pelz, Jonathan R. Lindner, Jan F. Stevens, Jacob Raber

**Affiliations:** ^1^Department of Behavioral Neuroscience, Oregon Health & Science University, Portland, OR, United States; ^2^Linus Pauling Institute, Oregon State University, Corvallis, OR, United States; ^3^Department of Animal & Rangeland Sciences, Oregon State University, Corvallis, OR, United States; ^4^Department of Pharmaceutical Sciences, College of Pharmacy, Oregon State University, Corvallis, OR, United States; ^5^Oregon Stem Cell Center and Department of Pediatrics, Oregon Health & Science University, Portland, OR, United States; ^6^Oregon National Primate Research Center, Oregon Health & Science University, Portland, OR, United States; ^7^Knight Cardiovascular Institute, Oregon Health & Science University, Portland, OR, United States; ^8^Division of Neuroscience ONPRC, Departments of Neurology and Radiation Medicine, Oregon Health & Science University, Portland, OR, United States

**Keywords:** proton irradiation, hippocampus, left ventricle, integrated epigenetic metabolomics analysis, radiation biomarkers, Parkinson's disease

## Abstract

In this study, an untargeted metabolomics approach was used to assess the effects of proton irradiation (1 Gy of 150 MeV) on the metabolome and DNA methylation pattern in the murine hippocampus and left ventricle of the heart 22 weeks following exposure using an integrated metabolomics-DNA methylation analysis. The integrated metabolomics-DNA methylation analysis in both tissues revealed significant alterations in aminoacyl-tRNA biosynthesis, but the direction of change was tissue-dependent. Individual and total amino acid synthesis were downregulated in the left ventricle of proton-irradiated mice but were upregulated in the hippocampus of proton-irradiated mice. Amino acid tRNA synthetase methylation was mostly downregulated in the hippocampus of proton-irradiated mice, whereas no consistent methylation pattern was observed for amino acid tRNA synthetases in the left ventricle of proton-irradiated mice. Thus, proton irradiation causes long-term changes in the left ventricle and hippocampus in part through methylation-based epigenetic modifications. Integrated analysis of metabolomics and DNA methylation is a powerful approach to obtain converging evidence of pathways significantly affected. This in turn might identify biomarkers of the radiation response, help identify therapeutic targets, and assess the efficacy of mitigators directed at those targets to minimize, or even prevent detrimental long-term effects of proton irradiation on the heart and the brain.

## Introduction

Protons are a major component of galactic cosmic rays (GCR) and solar particle events (SPE) (Lloyd et al., 2018). Beyond the shielding of the earth's magnetic field, astronauts will be exposed to ionizing radiation (IR) from SPEs, consisting primarily of low to medium energy protons (< ~150 MeV) with smaller components of helium and heavy nuclei, and to IR from GCR (Wilson, 2000). Therefore, proton irradiation as part of the space radiation environment of astronauts during missions may pose a significant long-term hazard (Simonsen et al., 1993; Cucinotta et al., 2013). This radiation exposure is also pertinent to cancer patients treated with protons (ICoR Units, 2007; Merchant et al., 2008; Lukens et al., 2015; Lupu-Plesu et al., 2017). In particular, the brain is sensitive to detrimental effects of proton irradiation in humans following cancer treatment (Merchant et al., 2008; Armstrong, 2012). Studies in rodent models are consistent with these human data (Rabin et al., 2002; Shukitt-Hale et al., 2004; Sanchez et al., 2010). Within the brain, the hippocampus might be especially sensitive to effects of proton irradiation (Rabinow et al., 2006; Bellone et al., 2014; Rudobeck et al., 2014; Parihar et al., 2015; Raber et al., 2015; Sokolova et al., 2015; Impey et al., 2016).

Compared to the hippocampus, only a few studies have been reported on the effects of proton radiation on the heart in humans. Radiation therapy for cancer treatment can lead to cardiotoxicity, suggesting that the heart is also predisposed to the effects of radiation. Additionally, there is little evidence of effective cardiac biomarkers for detecting cardiotoxicity, highlighting a need for more research (Conway et al., 2015). If the effects of proton irradiation on the heart are similar to those reported following gamma irradiation in cancer patients (Russell et al., 2009; Weintraub et al., 2010), relative late effects are anticipated (Halle et al., 2010; Lancellotti et al., 2013), and individuals in the age range of astronauts might be particularly susceptible to these long-term effects (Lancellotti et al., 2013).

Using an unbiased genome-wide approach, we previously reported that the effects of proton irradiation on the hippocampus and left ventricle 22 weeks following exposure involve tissue-dependent changes in DNA methylation, a major epigenetic modification that can impact gene expression (Impey et al., 2016). These data support increased understanding of the effects of radiation on DNA methylation in both rodent models and human cell lines (Miousse et al., 2017). Recently, research has emphasized the strong link between DNA methylation and metabolism, as some metabolic pathways are controlled by methylation-based epigenetic modifications (Chiacchiera et al., 2013). Furthermore, co-substrates generated by cellular metabolism are necessary for enzymatic control of epigenetic changes. Such alterations may even lead to disease such as cancer (Kaelin and McKnight, 2013). Diet-induced obesity also leads to changes in the epigenetic response to ionizing radiation (Vares et al., 2014). Both epigenetic modifications and altered metabolism may thus modulate the effects of ionizing radiation. As both metabolism and DNA methylation interact, we hypothesized that integrating the two would emphasize the most relevant pathways.

Recently, we combined genome-wide DNA methylation with untargeted metabolomics for an integrated analysis of the hippocampus of human apolipoprotein E mice in a high-fat diet (HFD)-induced insulin resistance (IR) mouse model (Johnson et al., 2017). This analysis revealed converging evidence for the involvement of specific pathways. In the current study, we used an untargeted metabolomics approach to assess the effects of proton irradiation (1 Gy of 150 MeV) on the metabolome in murine hippocampus and left ventricle of the heart 22 weeks following exposure. Additionally, we integrated these novel metabolomics data with our previous DNA methylation analysis to further identify biological pathways most affected by proton irradiation.

## Materials and Methods

### Animals and Study Design

Six-month-old C57Bl6/J male mice (*n* = 9 mice) were purchased from Jackson Laboratories, Bar Harbor Maine. The mice were shipped from the Jackson Laboratories to Brookhaven National Laboratory (BNL), Upton, Long Island, New York. After accommodating to the housing facility at BNL for 1 week, the mice were transported to the NASA Space Radiation Laboratory (NSRL) on the BNL campus and irradiated with 1 Gy of 150 MeV protons or sham-irradiated. The mice were loaded into 8 × 3 × 3 cm plastic square enclosures. These enclosures were either placed in a foam fixture in the beam line and exposed to a rectangular beam of ~20 × 20 cm generated by the Booster accelerator at BNL and transferred to the experimental beam line at the NSRL facility or received sham irradiation for the same time as the irradiated mice (*n* = 4–5 mice/exposure condition; the mice were randomly assigned to the two groups). Dose calibration was performed to obtain the targeted dose. The week following the irradiation or sham irradiation, the mice were shipped to Oregon Health & Science University (OHSU) and group-housed under standard care for 22 weeks after which they were euthanized by cervical dislocation for tissue analyses. The hippocampus of one hemibrain and half of the left ventricle of nine mice were divided into separate tissues for DNA methylation analyses. The hippocampus of the other hemibrain and the other half of the left ventricle were processed for untargeted metabolomics. The researchers were blinded to the exposure condition until after completion of all DNA methylation and metabolomics data acquisition and analyses. All protocols were reviewed and approved by the Institutional Animal Care and Use Committees (IACUC) of BNL and OHSU and in compliance with all federal regulations. Animal experiments were carried out in accordance with the relevant guidelines and regulations.

### Metabolomics

Hippocampal and left ventricular tissues were taken from sham-irradiated and proton-irradiated mice. These tissues were dissected and homogenized in RIPA (500 μl). Metabolites were extracted from 100 μl of hippocampal homogenates and 100 μl of left ventricular homogenates and untargeted metabolomics was completed as described (Kirkwood et al., 2013). Briefly, a Shimadzu Nexera system was used to run high-pressure liquid chromatography coupled to a quadrupole time-of-flight-mass spectrometer (Sciex TripleTOF 5600). This was operated in data-dependent MS/MS (IDA) acquisition mode in both positive and negative ion mode (Kirkwood et al., 2012).

Metabolomics data were processed using Markerview and Peakview software (AB Sciex, Framingham, MA, U.S.A.). Metabolites were initially identified by comparisons with an in-house library consisting of 619 IROA standards (IROA Technologies, Bolton, MA, U.S.A.) and 30 other additional commercially available standards. Additional metabolites were individually identified based on MS isotopic pattern, accurate mass (mass error <5 ppm), MS/MS fragment ions, and matching with spectra in online databases, including Metlin, Human Metabolome Database (HMDB), and Lipidmaps.

To assess analytical variability compared to biological variation, we included four technical replicate analyses of a quality control (QC) sample, which was prepared by mixing 5-μl aliquots of all nine biological processed samples from the proton-irradiated and sham-irradiated mice, and visualized the non-centered, Pareto scaled data by Principal Component Analysis of hippocampal tissue (Supplementary Figure 1A), and ventricle tissue (Supplementary Figure 1B). These data show that the variation in the technical replicates is negligible compared to the biological variation.

### DNA Methylation

DNA methylation was performed as described (Impey et al., 2016, 2017). Briefly, DNA was isolated from the left ventricle and hippocampus in the same mice processed for metabolomics analyses. Antibodies against 5-methylcytosine (5 mC) and 5-hydroxymethylcytosine (5 hmC) that do not cross react were used to immunoprecipitate DNA fragments (DIP). Eight pools of tissues (2 × 2 pools of hippocampal tissues and 2 × 2 pools of left ventricle tissues, or 2 pools/tissue/radiation condition) were used. For DIP-Seq library preparation, RNAse-treated DNA was isolated using the Qiagen Allprep DNA/RNA protocol, involving “randomly” fragmenting dsDNA fragmentase (NEB), polishing using the DNA terminator end repair kit (Lucigen), and adding an “A” base to the 3′ end of DNA fragments using Klenow exo- (Epicenter). The genomic DNA was ligated to un-methylated HT TrueSeq indexed adapters, purified, denatured, resuspended in 100 μl of DIP IP buffer, and immunoprecipitated, and the eluted DNA was purified and subjected to limited amplification (~15 cycles).

Illumina high-throughput genomic sequencing of the immunoprecipitated meDIP DNA (DIP-Seq) on a HiSeq2000 platform was used to identify differentially methylated regions and associated genes throughout the genome. We generated a bioinformatic pipeline to map and annotate DIP-Seq sequencing and statistical algorithms that allow for the identification of differentially methylated regions in DIP-Seq data sets.

### Statistical Analyses

To identify pathways (and associated metabolites) that are affected by radiation, we used a multi-step statistical analysis approach based in part on previous analyses (Xia and Wishart, 2010; Kirkwood et al., 2013). In the first exploratory phase, we used an untargeted metabolomics approach and determined for the metabolomics data the top 5 pathways with at least 3 identified metabolites that were altered by radiation treatment. Signal intensities of identified metabolites were annotated with HMDB numbers. Annotated, non-transformed signal intensities were Pareto-scaled in MetaboAnalyst 4.0 (Chong et al., 2018). For pathway analysis using known *Mus musculus* KEGG pathways in MetaboAnalyst, only metabolites with HMDB number were included. For multi-comparison adjustment, false-discovery rates (FDR) are reported (Benjamini and Hochberg, 1995). The R scripts generated are included as Supplementary File 1.

For the integrated DNA methylation metabolomics analysis, we first reanalyzed the DNA methylation data of our original publication (Impey et al., 2016). Briefly, regions of methylation enrichment (i.e., 5 hmC regions) were merged. Treatment group differences were determined by negative binomial tests. Gene ontology analyses involved the bioconductor Goseq package, which adjusts for RNA-Seq length bias artifacts. To confirm that the re-analyzed DNA methylation did not differ from those in our original publication (Impey et al., 2016), pathway data were visualized using R/Bioconductor (Roswell Park Cancer Institute, Buffalo, NY) or Cytoscape software (Shannon et al., 2003). The reanalyzed data gave similar results as our original publication (Impey et al., 2016) and are posted on the NIH Gene Expression Omnibus (GEO) web site (accession number: GSE132588). The C source code will be provided upon request. DNA methylation data were then merged with metabolomics data in MetaboAnalyst using only methylation regions with treatment group differences with a raw *p*-value of < 0.01. The R scripts used for MetaboAnalyst are included as a .zip file as Supplementary File 2. In short, pathway analyses and enrichment analyses were set to “hypergeometric test,” topology analysis set to “degree centrality,” and “gene-metabolite pathways” were chosen, as described (Johnson et al., 2017). After FDR adjustment, the aminoacyl-tRNA biosynthesis pathway was identified as the only significantly altered pathway.

In the second, targeted phase, in which we show that radiation altered metabolite abundance as well as methylation of the amino-tRNA biosynthesis pathway, we focused on the 15 identified amino acids of the amino-tRNA biosynthesis pathways as well as the methylation status of their tRNA synthetases and did not adjust for multiple comparisons. Non-transformed signal intensities of the 15 amino acids of the aminoacyl-tRNA biosysnthesis pathway were Pareto-scaled in MetaboAnalyst and the effect of radiation was visually examined using partial least squares–discriminant analysis (PLS-DA) in MetaboAnalyst. The visual examination identified one mouse sample (number 11) as an outlier. Therefore, the statistical comparison between sham vs. proton-irradiated mice using the non-parametric Kruskal-Wallis test was done once with and once without number 11. In addition, we used an unpaired student's test to compare sham-irradiated vs. proton-irradiated methylation patterns of amino acid tRNA synthetases. All statistical tests were two-sided. Significance was set at *p* ≤ 0.05 and a statistical tendency at *p* < 0.10 unless otherwise noted.

Figures were generated using MetaboAnalyst 4.0 (Quebec, Canada), GraphPad Prism v6 (La Jolla, CA), and GIMP (http://GIMP.org). To provide a comparable scale, changes in individual and total amino acids are shown as % changes from sham-irradiated mice (median proton-irradiated mice/median sham-irradiated mice in % −100) in **Figure 3**.

## Results

### Metabolite Identification

Using untargeted metabolomics, we detected and semi-quantified mass spectral features in hippocampal, and left ventricle heart tissues of proton irradiated and sham-irradiated mice. Using accurate mass and MS/MS and isotope ratio patterns, we assigned 127 features to metabolite identities using online database searching, 78 of which were confirmed by our in-house library of metabolite standards using PeakView software (Supplementary Tables 1, 2). There were a small proportion of metabolites that were identified in both the positive and negative ion mode. For these, we evaluated them using the student's *t*-test and chose the ion mode data for each metabolite found in duplicate based on its abundance and levels of significance.

### Metabolomics Analysis Hippocampus

**Figure 1:**
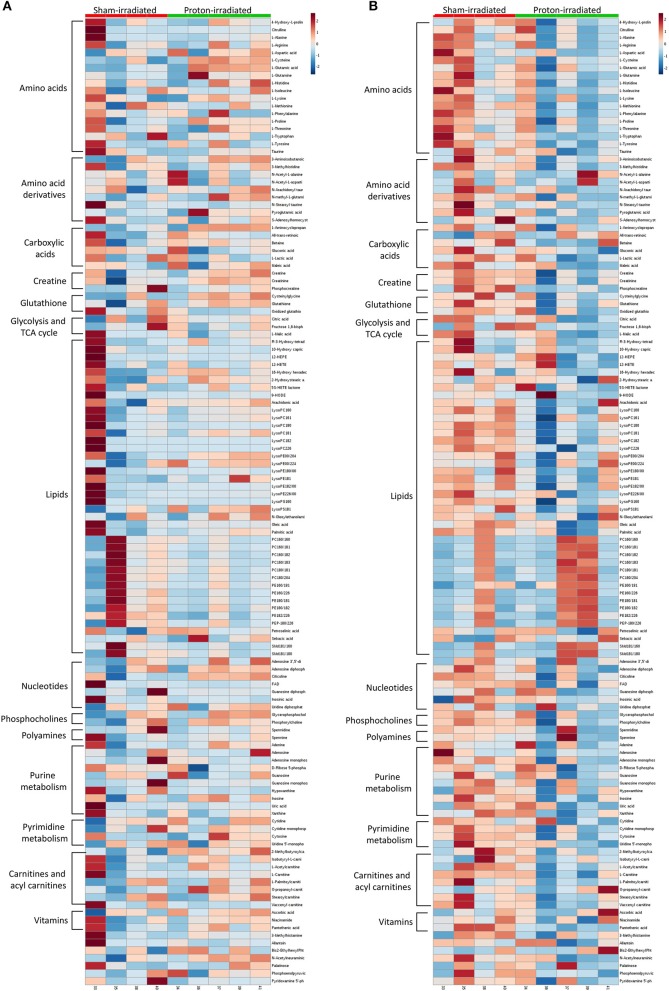
Heatmaps of metabolomics analyses of the hippocampus **(A)** and left ventricle **(B)** 22 weeks following proton and sham irradiation.

**Figure 2:**
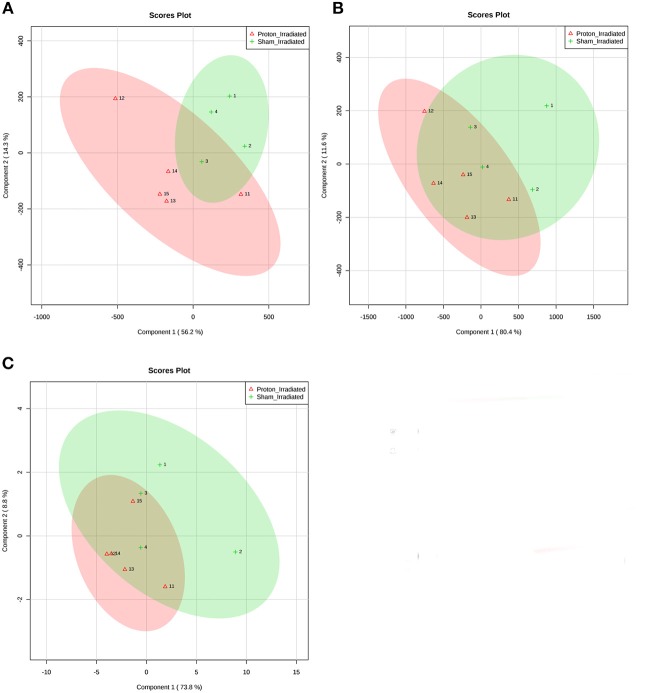
Partial least squares discriminant analysis (PLS-DA) of **(A)** hippocampal tissue, **(B)** left ventricular tissue, and **(C)** the ratio of left ventricular tissue and hippocampal tissue discriminates between sham- and proton-irradiated mice. Signal intensities of 15 individual amino acids were scaled (Pareto scaling) and then analyzed using PLS-DA. The ellipses were drawn at 95% CI of normal distribution for a given group. Loading plots illustrate the proportion of variation accounted for by each component. Each number represents one mouse (1–4 for sham-irradiated mice, and 11–15 for proton-irradiated mice).

**Figure 3:**
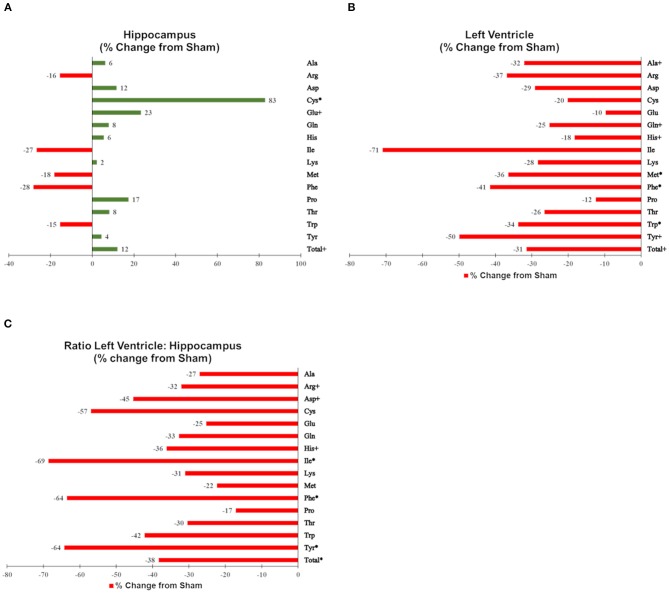
Effects of proton irradiation on signal intensities of individual and total amino acids in **(A)** hippocampal tissue, **(B)** left ventricular tissue, and **(C)** the ratio of left ventricular tissue and hippocampal tissue in sham-, and proton-irradiated mice. Results are shown as % changes from sham-irradiated mice (median proton-irradiated mice/median sham-irradiated mice in % −100). Amino acids with a ^*^ signify a *p* ≤ 0.05 and amino acids with a + signify a 0.05 < *p* ≤ 0.10.

Of the 52 identified KEGG pathways, no pathways were significantly altered by proton irradiation in hippocampal tissue, although several pathways trended toward significance (see Supplementary Table 3 for top 5 of 15 pathways with at least 3 identified metabolites). The heat map analysis of individual metabolites is visualized in Figure 1A. The only pathway that ranked in the top 5 pathways for each tissue and each analysis (Supplementary Tables 3, 4) was the aminoacyl-tRNA biosynthesis pathway. Based on these findings, we focused our further statistical analysis on the aminoacyl-tRNA biosynthesis pathway. A PLS-DA analysis visualized that the 15 identified amino acids of the aminoacyl-tRNA biosynthesis pathway could differentiate between sham- and proton-irradiated mice (Figure 2A). The first principal component, which differentiated all but one proton-irradiated mouse (number 11), accounted for 56.2% of the variability. Next, we examined each of 15 identified amino acids individually or as a sum. Using the non-parametric Wilcoxon Rank Sum test, cysteine was 83% higher in proton- than sham-irradiated mice (*p* = 0.01), and glutamate tended to be 23% higher in proton- than sham-irradiated mice (*p* = 0.08). The sum of all 15 amino acids tended to be 12% higher in proton- than sham-irradiated mice (*p* = 0.08; Figure 3A). After exclusion of mouse number 11, mice of the two groups differed in the levels of cysteine, glutamate, and total amino acids, with higher values in the proton-irradiated mice and a *p*-value of 0.02.

### Metabolomics Analysis Left Ventricle of the Heart

Of the 52 identified KEGG pathways, no pathways were significantly altered following FDR adjustment by proton irradiation in the left ventricle of the heart (see Supplementary Table 3 for top 5 of 15 pathways with at least 3 identified metabolites). The heat map analysis of individual metabolites is visualized in Figure 1B. A PLS-DA analysis showed that the 15 identified amino acids of the aminoacyl-tRNA biosynthesis pathway differentiated between sham- and proton-irradiated mice (Figure 2B). The first principal component, which differentiated all but one proton-irradiated mouse (number 11), accounted for 80.4% of the variability and the second another 11.6%, indicating an even greater impact of proton irradiation on the left ventricle than on the hippocampus. In contrast to the hippocampus, protein irradiation consistently decreased protein values for all 15 amino acids. Significant decreases were observed for 3 of 15 amino acids, namely phenylalanine (−41%; *p* = 0.05), tryptophan (−34%; *p* = 0.05), and methionine (−36%; *p* = 0.05), and a tendency for another 4 amino acids, namely alanine (−32%; *p* = 0.09), glutamine (−25%; *p* = 0.09), histidine (−18%; *p* = 0.09), and tyrosine (−50%; *p* = 0.09). The sum of all 15 amino acids tended to be 31% lower in proton- vs. sham-irradiated mice (*p* = 0.09; Figure 3B). As was for the hippocampus, our analysis only misclassified mouse number 11. After exclusion of number 11, mice of the two groups perfectly separated for 4 of 15 amino acids (histidine, phenylalanine, tryptophan, and methionine) with lower values for proton-than sham-irradiated mice and a *p*-value of 0.02. One misclassification and a *p*-value of 0.04 was achieved for total amino acids and another 3 amino acids (glutamine, isoleucine, and tyrosine).

### Comparison Between Metabolites in the Left Ventricle of the Heart and Those in Hippocampus

To evaluate whether proton irradiation affected the aminoacyl-tRNA biosynthesis pathway differently in the left ventricle and the hippocampus, we calculated the ratio of the left ventricle and hippocampus for each amino acid. Consistent with the pattern in the left ventricle, the ratio for each amino acid went down following proton irradiation. PLS-DA analysis with those 15 amino acid ratios could separate sham- and proton-irradiated mice (Figure 2C). Significant decreases were observed for 3 of 15 amino acids, namely phenylalanine, isoleucine, and tyrosine (all *p* = 0.05), and a tendency for another 3 amino acids, namely arginine, aspartate, and histidine (all *p* = 0.09; Figure 3C). The ratio for the sum of all 15 amino acids was lower in proton- than sham-irradiated mice (*p* = 0.05). The only misclassified mouse was number 11. After exclusion of number 11, the mice of the two groups perfectly separated for total amino acid ratio and 3 amino acid ratios (arginine, histidine, and isoleucine) with lower values for proton-irradiated than sham-irradiated mice and a *p*-value of 0.02. One misclassification and a *p*-value of 0.04 was achieved for another 4 amino acids (glutamate, glutamine, phenylalanine, and tyrosine).

### Integrated Metabolomics-DNA Methylation Analysis of the Hippocampus

We used our updated bioinformatic pipeline to reanalyze the left ventricle and hippocampal DNA methylation reported in the original publication [(Impey et al., 2016); see GEO web site accession number: GSE132588]. Next, we integrated DNA methylation data and metabolomic data using the joint pathway analysis function of MetaboAnalyst, which compared alterations in DNA methylation profiles and metabolic pathways using KEGG annotations as the reference frame.

The integrated analysis of hippocampal tissue revealed significant alterations following FDR adjustment in the aminoacyl-tRNA biosynthesis pathway (Supplementary Table 4). Of the 15 amino acids, we identified tRNA synthetase methylation of 9 amino acids. Proton irradiation resulted in significantly lower values in tRNA synthetase methylation of 44% (4 of 9) of amino acids, namely threonine (−80%; *p* = 0.005), tryptophan (−79%; *p* = 0.007), phenylalanine (−75%; *p* = 0.01), and isoleucine (−72%; *p* = 0.02), and a tendency for arginine (−70%; *p* = 0.07). The pattern of down-regulation was consistent as only 1 of 9 amino acid tRNA synthetases had higher methylation values (cysteine: +77%; *p* = 0.36) in proton- than sham-irradiated mice (results not shown).

### Integrated Metabolomics-DNA Methylation Analysis of the Left Ventricle

The integrated analysis of left ventricular tissue also revealed significant alterations in aminoacyl-tRNA biosynthesis (Supplementary Table 4). Next, we combined our metabolomics and DNA methylation results for the aminoacyl-tRNA biosynthesis pathway. Of the 15 amino acids, we identified tRNA synthetase methylation of 3 amino acids. Proton irradiation resulted in significantly higher values in tRNA synthetase methylation of phenylalanine (+562%; *p* = 0.02), whereas isoleucine and threonine were not significantly affected. However, we observed significantly lower values in tRNA synthetase methylation of asparagine (−92%; *p* = 0.002). No consistent methylation pattern was observed comparing proton- vs. sham-irradiated mice.

## Discussion

The data of the current study support the value of metabolomics and especially integrated analyses of metabolomics and DNA methylation to identify long-term changes in distinct tissues following whole body radiation. Below we first discuss the metabolomics analysis by itself, followed by the integrated metabolomics DNA methylation analysis.

Using untargeted metabolomics analysis by itself, the current study was not able to identify pathways that were significantly altered by proton irradiation in the hippocampus and the left ventricle of the heart using FDR adjustment. However, the pathway analysis revealed aminoacyl-tRNA biosynthesis as the most promising pathway to further pursue for targeted analysis. Previous work in metabolic profiling of urine samples from C57BL/6J mice 4 h following proton irradiation (0.5 and 2 Gy of 1 GeV) revealed profound alterations in energy metabolism, amino acid metabolism, and purine and pyrimidine metabolism (Laiakis et al., 2015). Taken with our findings, this suggests that proton irradiation may cause early systemic changes in biofluids, with long-lasting changes in tissues. Thus, even a single proton irradiation event can lead to long-term changes.

Aminoacyl-tRNA biosynthesis, key for protein synthesis including the accuracy of translation (Ibba and Söll, 2004), was significantly altered (FDR adjusted) by proton irradiation in the hippocampus and left ventricle as demonstrated by the integrated metabolomics-DNA methylation analysis. Interestingly, aminoacyl-tRNA biosynthesis was recently identified as one of two metabolic pathways most strongly associated with left ventricular diastolic dysfunction, and consistent over a 5 year period, in 570 randomly recruited people (Zhang et al., 2013). The downregulation of metabolites in this pathway in the left ventricle of proton-irradiated mice is especially concerning as deficiency in tRNA synthetase editing activity can cause cardioproteinopathy (Liu et al., 2014). Mutations in aminoacyl-tRNA synthetases have been linked to several disease conditions, including cancer and various neurodegenerative conditions like Parkinson's Disease (Park et al., 2008; Sauter et al., 2015; Kapur et al., 2017; Meyer-Schuman and Antonellis, 2017). Hence further analysis is warranted considering its role in cardiovascular function.

In summary, proton irradiation causes long-term changes in the left ventricle and hippocampus. The aminoacyl tRNA biosynthesis pathway might be especially valuable as a biomarker of the proton radiation response. Future studies should address to what extent this pathway could be targeted to attenuate detrimental long-term effects of proton irradiation on the heart and the brain. Thus, integrated analysis of metabolomics and DNA methylation is a powerful approach to obtain converging evidence of pathways significantly affected by proton irradiation. Our findings also suggest the importance of assessing tissue-specific compensatory changes. Such tissue-dependent compensatory directional changes will likely depend on the time interval after radiation exposure. A limitation of our study is that we included only behaviorally naïve mice, as behavioral testing would be expected to affect DNA methylation and metabolic pathways and we wanted to assess the effects of proton irradiation by itself. As permutation and cross-validation tests require relative large data sets and the statistical power of ranking tests is limited, we did not adjust for multiple comparisons in the targeted analysis of the 15 identified amino acids of the amino-tRNA biosynthesis pathways as well as the methylation status of their tRNA synthetases. Therefore, future studies are warranted to compare radiation responses at distinct intervals following radiation exposure and involving larger group sizes and comparisons between behaviorally naïve and behaviorally tested mice following proton irradiation.

## Ethics Statement

This study was carried out in accordance with the recommendations of the OHSU and BNL IACUC committee. The protocol was approved by the OHSU and BNL IACUC committees.

## Author Contributions

SI, JS, and JR conceived and planned this study. ET, RH, GB, JC, CP, and SI analyzed the data. All authors discussed the results and contributed to the final manuscript.

### Conflict of Interest Statement

The authors declare that the research was conducted in the absence of any commercial or financial relationships that could be construed as a potential conflict of interest.
