# Supplementary material for: Integrated Metabolomics-DNA Methylation Analysis Reveals Significant Long-Term Tissue-Dependent Directional Alterations in Aminoacyl-tRNA Biosynthesis in the Left Ventricle of the Heart and Hippocampus Following Proton Irradiation
Source: Front Mol Biosci. 2019 Sep 10;6:77. doi: 10.3389/fmolb.2019.00077 (PMC6746933; doi:10.3389/fmolb.2019.00077)
Supplement: Supplementary file 3 [file Table_3.DOCX]

**Supplementary Table 3**. Top 5 pathways with at least 3 identified metabolites that were affected by irradiation in the hippocampus and the ventricle.^1^

|  | Compounds | | Comparison | |
| --- | --- | --- | --- | --- |
| Top 5 Pathways | Total | Hit | Raw P | FDR |
| *Hippocampus:* |  |  |  |  |
| His metabolism | 15 | 4 | 0.06 | 0.71 |
| Nitrogen metabolism | 9 | 3 | 0.15 | 0.71 |
| Aminoacyl tRNA biosynthesis | 69 | 15 | 0.19 | 0.71 |
| Glutathione metabolism | 26 | 8 | 0.36 | 0.71 |
| Gly/Ser/Thr metabolism | 31 | 4 | 0.37 | 0.71 |
| *Left Ventricle:* |  |  |  |  |
| Cys/Met metabolism | 27 | 3 | 0.01 | 0.25 |
| Glycerophospholipid metabolism | 30 | 6 | 0.05 | 0.25 |
| Gly/Ser/Thr metabolism | 31 | 4 | 0.09 | 0.25 |
| Aminoacyl tRNA biosynthesis | 69 | 15 | 0.10 | 0.25 |
| Arg/Pro metabolism | 44 | 10 | 0.11 | 0.25 |

^1^ Total compounds are the number of known metabolites in the respective KEGG pathways. Hits are the number of identified metabolites in this study that are associated with the pathway. Raw P refers to unadjusted p-value. FDR refers to False Discovery Rate.
