# Supplementary material for: Integrated Metabolomics-DNA Methylation Analysis Reveals Significant Long-Term Tissue-Dependent Directional Alterations in Aminoacyl-tRNA Biosynthesis in the Left Ventricle of the Heart and Hippocampus Following Proton Irradiation
Source: Front Mol Biosci. 2019 Sep 10;6:77. doi: 10.3389/fmolb.2019.00077 (PMC6746933; doi:10.3389/fmolb.2019.00077)
Supplement: Supplementary file 4 [file Table_4.DOCX]

**Supplementary Table 4**. Top 5 pathways with at least 3 identified metabolites that were affected by irradiation in the hippocampus and the ventricle in the integrated analysis.^1^

|  | Compounds | | Comparison | |
| --- | --- | --- | --- | --- |
| Top 5 Pathways | Total | Hits | Raw P | FDR |
| *Hippocampus:* |  |  |  |  |
| **Aminoacyl tRNA biosynthesis** | **91** | **17** | **0.001** | **0.03** |
| Arg/Pro metabolism | 102 | 14 | 0.04 | 0.52 |
| Glutathione metabolism | 79 | 11 | 0.06 | 0.52 |
| Phe/Tyr/Trp metabolism | 12 | 3 | 0.07 | 0.52 |
| Sulfur metabolism | 16 | 3 | 0.15 | 0.82 |
| *Left Ventricle:* |  |  |  |  |
| **Aminoacyl tRNA biosynthesis** | **91** | **16** | **0.0003** | **0.006** |
| His metabolism | 45 | 6 | 0.08 | 0.65 |
| Arg/Pro metabolism | 102 | 11 | 0.08 | 0.65 |
| Glutathione metabolism | 79 | 8 | 0.17 | 0.92 |
| Ala/Asp/Glu metabolism | 58 | 6 | 0.20 | 0.92 |

^1^ Total compounds are the number of known metabolites in the respective KEGG pathways. Hits are the number of identified metabolites in this study that are associated with the pathway. Raw P refers to unadjusted p-value. FDR refers to False Discovery Rate.
