# Supplementary figures and images for: Integrated Metabolomics-DNA Methylation Analysis Reveals Significant Long-Term Tissue-Dependent Directional Alterations in Aminoacyl-tRNA Biosynthesis in the Left Ventricle of the Heart and Hippocampus Following Proton Irradiation
Source: Front Mol Biosci. 2019 Sep 10;6:77. doi: 10.3389/fmolb.2019.00077 (PMC6746933; doi:10.3389/fmolb.2019.00077)

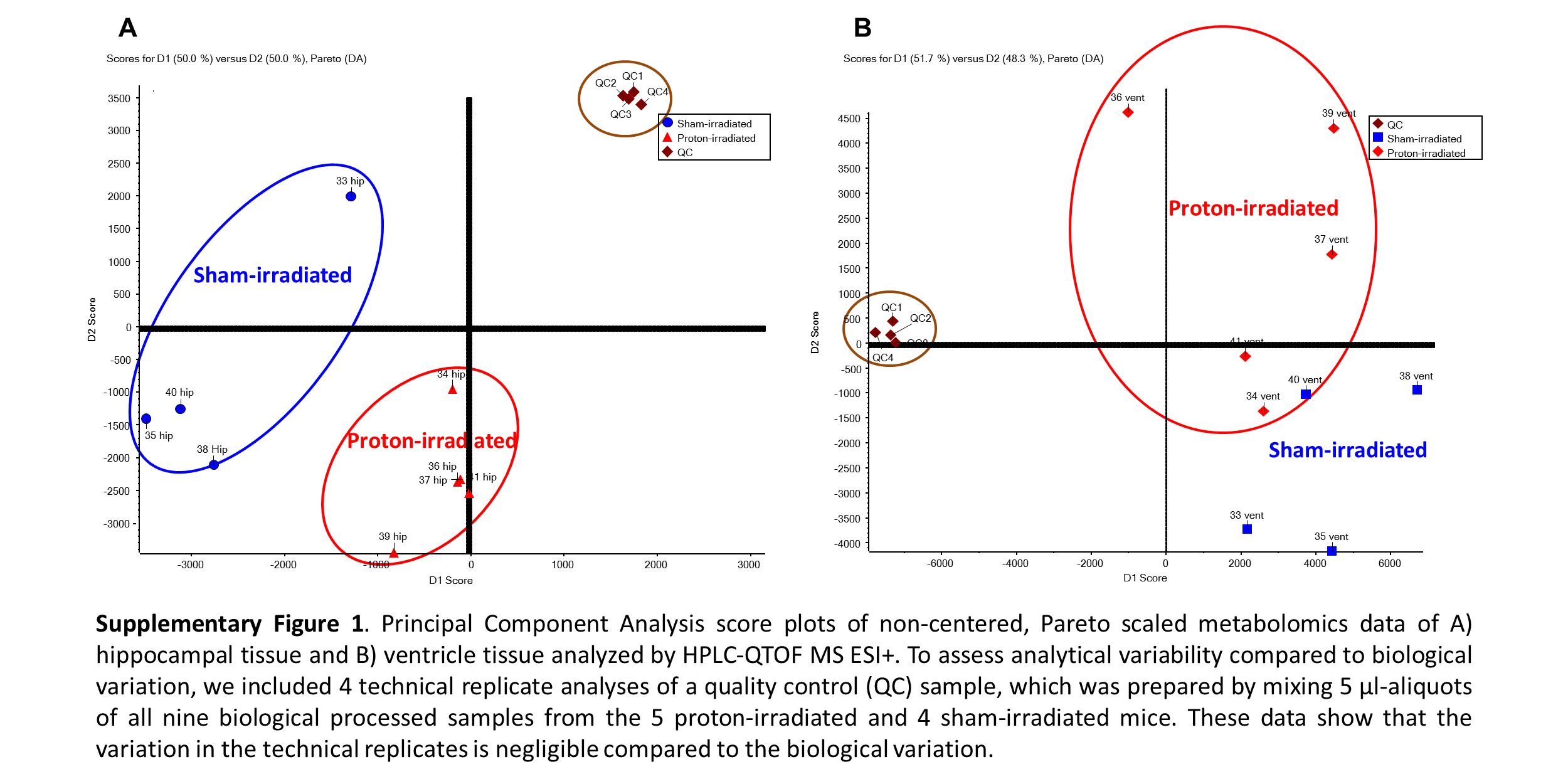

Supplement: Supplementary file 7 [file Image_1.TIFF]
